# VEXAS Syndrome Beyond UBA1: Genetic Architecture and the Role of Co-Occurring Somatic Mutations—A Focused Review

**DOI:** 10.3390/genes17060711

**Published:** 2026-06-20

**Authors:** Konstantin N. Konstantinov, Nikifor K. Konstantinov, Vijayalakshmi Kumar

**Affiliations:** 1 Medicine Service, Section of Rheumatology, Raymond G. Murphy VA Medical Center, Albuquerque, NM 87108, USA; vijayalakshmi.kumar@va.gov; 2 Department of Medicine, University of New Mexico, Albuquerque, NM 87131, USA; 3 Department of Dermatology, University of New Mexico, Albuquerque, NM 87131, USA

**Keywords:** VEXAS syndrome, *UBA1*, DNMT3A, clonal hematopoiesis, autoinflammatory disease, somatic mutations

## Abstract

Vacuoles, E1 enzyme, X-linked, autoinflammatory, somatic (VEXAS) syndrome is an adult-onset inflammatory disorder caused by acquired mutations in *UBA1*, the gene encoding the primary ubiquitin-activating enzyme. The recognition of VEXAS has transformed the current understanding of autoinflammatory disease by demonstrating that somatic alterations arising within hematopoietic stem cells can precipitate severe, multisystem inflammation in later life. While pathogenic *UBA1* variants are essential to disease pathogenesis, many affected individuals also harbor additional somatic mutations associated with clonal hematopoiesis, most commonly involving *DNMT3A* and *TET2*. These concurrent mutations may contribute to clonal architecture; however, their independent impact on inflammatory phenotype and hematologic manifestations remains incompletely defined. Emerging evidence suggests that co-occurring clonal hematopoiesis mutations may be independently associated with poorer overall survival, though their causal role remains unestablished. This review examines the evolving genetic framework of VEXAS syndrome, emphasizing *UBA1* as the obligate driver mutation while reviewing current evidence regarding non-Met41 *UBA1* variants and co-occurring somatic mutations.

## 1. Introduction

VEXAS syndrome is an acquired autoinflammatory condition that typically presents in later adulthood, particularly in men over 50 years of age, and is characterized by systemic inflammation, hematologic abnormalities, and distinctive cytoplasmic vacuolization within bone marrow precursors of both myeloid and erythroid cell lineages [1,2,3,4,5]. Unlike inherited autoinflammatory disorders, VEXAS arises from acquired somatic mutations in hematopoietic progenitor cells and is closely linked to clonal myeloid disease, together driving systemic inflammatory pathology [1,2,3,4,5]. Since its initial description, VEXAS has been increasingly recognized across diverse clinical disciplines, including but not limited to rheumatology, hematology, dermatology, and pulmonary medicine.

Mounting evidence has demonstrated that the molecular landscape of VEXAS extends beyond mutations in *UBA1*. The most common pathogenic variants are p.Met41Thr (c.122T>C), p.Met41Val (c.121A>G), and p.Met41Leu (c.121A>C). With the growing use of myeloid-focused next-generation sequencing, it has become evident that *UBA1* variants frequently coexist with other somatic mutations commonly associated with clonal hematopoiesis [2]. Clonal hematopoiesis (CH) is an age-related process in which somatic mutations confer a competitive advantage to hematopoietic stem cells without immediate malignant transformation. CH is associated with increased risk of all-cause mortality, coronary heart disease, and ischemic stroke [6]. It is also associated with autoimmune and autoinflammatory disease [2]. In VEXAS syndrome, clonal hematopoiesis is highly prevalent and frequently coexists with *UBA1* mutations [2].

Clonal hematopoiesis of indeterminate potential (CHIP) mutations (*DNMT3A*, *TET2*, and *ASXL1*) are known to promote inflammation and impair apoptosis, creating a shared molecular basis between hematological malignancies and inflammatory diseases [2,5,6,7,8,9,10,11]. Among these, mutations in *DNMT3A* and *TET2* are frequently identified [2]. Their presence supports the concept that VEXAS exists within the broader spectrum of age-related CHIP rather than as an isolated monogenic entity.

Understanding how these mutations interact is essential for enhancing diagnostic precision, fine-tuning prognostic assessments, and advancing treatment approaches.

This focused review was based on a targeted search of PubMed/MEDLINE and Google Scholar for English-language articles published from 2020 through January 2026. Search terms included “VEXAS syndrome,” “*UBA1*,” “Met41,” “non-Met41,” “DNMT3A,” *“TET2*,” “*ASXL1,*” “clonal hematopoiesis,” “CHIP,” “myelodysplastic syndrome,” “somatic mutations,” “genotype phenotype,” and “treatment.” Additional sources were identified from the reference lists of key articles and recent guidelines or consensus documents. Studies were selected for relevance to VEXAS pathogenesis, co-occurring somatic mutations, genotype–phenotype correlations, prognostic implications, molecular testing, and therapeutic strategies.

## 2. Clinical Vignette: *DNMT3A*-Associated Clonal Hematopoiesis Preceding VEXAS Diagnosis

A 69-year-old male was evaluated in our rheumatology clinic for suspected relapsing polychondritis. His history was notable for retinal hemorrhage, retinal vein occlusion, episodic upper eyelid swelling, bilateral unprovoked deep vein thromboses, progressive hearing loss, and pulmonary embolism despite anticoagulation.

Two months before presentation, he developed painful swelling of the right ear, followed by involvement of the left ear, refractory to multiple antibiotic courses. Laboratory evaluation demonstrated elevated inflammatory markers, leukopenia (3.8 × 10^9^/L), macrocytic anemia (hemoglobin 8.7 g/dL; mean corpuscular volume 107 fL), mild transaminitis, and negative autoimmune and infectious serologies. Bone marrow biopsy revealed hypercellular marrow with megakaryocytic dysplasia and no increase in blasts. Next-generation sequencing identified a *DNMT3A* mutation (c.2446C>T), variant allele frequency (VAF) 40%, with normal cytogenetics, leading to a diagnosis of low-risk myelodysplastic syndrome (MDS). The patient initially responded to glucocorticoids but failed methotrexate. A trial of decitabine was complicated by severe leukopenia and pneumonia. Over the next 14 months, he developed recurrent biopsy-proven urticarial leukocytoclastic vasculitis, transfusion dependence, retinal branch vein occlusion, ischemic stroke, and progressive cytopenias. Subsequent review of the bone marrow histopathology demonstrated cytoplasmic vacuolization, and next-generation sequencing of whole blood identified a somatic *UBA1* variant (c.121A>C; p.Met41Leu), VAF 38%, establishing the diagnosis of VEXAS syndrome.

Treatment with ruxolitinib resulted in significant improvement of inflammatory symptoms, though transfusion dependence persisted. Over time, therapeutic efficacy diminished, and the patient transitioned to hospice and supportive care. This case illustrates how *DNMT3A*-driven clonal hematopoiesis may precede recognition of VEXAS syndrome, contributing to diagnostic delay.

## 3. *UBA1*: The Driver Gene in VEXAS Syndrome

The *UBA1* gene encodes the ubiquitin-activating enzyme E1, a crucial upstream regulator of the ubiquitin–proteasome system. Pathogenic mutations in VEXAS selectively impair the cytoplasmic isoform (UBA1b), resulting in defective ubiquitination within myeloid cells while leaving the nuclear isoform relatively preserved [1]. As shown by prior studies and summarized by Groarke et al., pathogenic M41 mutations specifically disrupt translation initiation of the cytoplasmic *UBA1*b isoform while preserving nuclear *UBA1*a, providing the mechanistic basis for myeloid-restricted disease [5]. Most disease-causing variants affect methionine-41 (p.Met41Thr, p.Met41Val, p.Met41Leu), although additional missense and splice-site variants have broadened the genotypic spectrum of VEXAS [1,2,3,4,5]. The study by Sakuma et al. identified novel *UBA1* variants beyond the canonical methionine-41 (M41) mutations in VEXAS syndrome using whole-genome transcriptome sequencing in a large cohort of hematological malignancies [12]. A subsequent study by Sakuma et al. has shown that *UBA1*^non-M41^ variants can be associated with distinct biologic and hematologic features, including greater co-mutation complexity and different patterns of marrow involvement, although current genotype–phenotype correlations remain preliminary and should be interpreted cautiously [13]. *UBA1* mutations are somatic, largely myeloid-restricted, and not inherited. Dysfunction in ubiquitin activation leads to dysregulated innate immune signaling, cytokine excess, and the characteristic cytoplasmic vacuolization of erythroid and myeloid precursors observed in bone marrow. *UBA1* mutations drive VEXAS pathophysiology through four interconnected mechanisms: (1) inflammation via unfolded protein response (UPR)/endoplasmic reticulum (ER) stress activation and upregulation of IL-1, IL-6, IL-18, TNF, and interferon signaling; (2) cytopenias from selective toxicity to erythrocytes and lymphoid cells (causing macrocytic anemia and lymphopenia) while sparing myeloid cells; (3) clonality through cytokine-mediated suppression of wild-type cells, allowing UBA1-mutant hematopoietic stem and progenitor cells (HSPCs) dominance; and (4) oncogenicity, with MDS occurring in 18–55% of cases depending on diagnostic criteria. More recently, atypical and *UBA1*^non-M41^ variants have been associated with hematologic neoplasms beyond MDS, including lymphoproliferative disorders and acute myeloid leukemia (AML); however, these cases appear to retain myeloid skewing, with the absence of the mutation in lymphoid cells [1,5,12,13,14]. These characteristics identify *UBA1* as the essential and sufficient genetic mutation responsible for VEXAS syndrome.

In addition, recent studies have expanded our understanding of how somatic *UBA1* mutations in VEXAS syndrome drive systemic autoinflammation and hematopoietic clonal expansion. In a humanized mouse model, Molteni et al. demonstrated that mutant stem cells create a pro-inflammatory bone marrow milieu that “poisons” wild-type hematopoiesis, favoring mutant clone survival and expansion [15]. Furthermore, the degree of proteotoxic stress and unfolded protein response activation described in VEXAS may confer survival resilience to mutant clones, linking inflammation to selective clonal expansion [16]. This mechanism raises a key question: should clinicians target the clonal process or the inflammatory response—or are they, in fact, inseparable?

## 4. *DNMT3A* in VEXAS Syndrome

Among co-occurring somatic mutations, *DNMT3A* has emerged as one of the most frequently reported in VEXAS syndrome. *DNMT3A* encodes DNA methyltransferase 3A, an enzyme involved in de novo DNA methylation and epigenetic regulation of hematopoiesis. Because *DNMT3A* mutations are also among the most common drivers of age-related clonal hematopoiesis and myeloid neoplasia, their presence in VEXAS likely reflects, at least in part, the broader clonal hematopoietic background in which *UBA1*-mutant disease arises [5,6,7,8,9,10].

Clonal studies indicate that *DNMT3A* and other CH mutations may arise either before acquisition of *UBA1*, as subclones or in independent clones, supporting dynamic clonal evolution rather than a fixed hierarchical sequence [2]. Although no validated VAF thresholds exist for clinical decision-making in VEXAS syndrome, *UBA1* mutations are typically detected at moderate-to-high VAF in myeloid cells, consistent with clonal involvement of hematopoietic progenitors [1,2,3,5]. By contrast, *DNMT3A* and other clonal hematopoiesis-associated mutations may be present at variable clone sizes. Importantly, Gutierrez-Rodrigues et al. demonstrated that typical clonal hematopoiesis mutations, including *DNMT3A* and *TET2,* were not associated with specific inflammatory or hematologic manifestations in VEXAS patients [2]. Critically, while co-mutations do not appear to drive disease heterogeneity, they carry prognostic significance. Gutierrez-Rodrigues et al. found that typical clonal hematopoiesis mutations were independently correlated with poor overall survival [2].

## 5. Other Co-Occurring Somatic Mutations in VEXAS

*TET2*, another regulator of epigenetic programming, is also found in VEXAS and may occur alongside or even before *UBA1* mutations [2,10,11]. Beyond *DNMT3A* and *TET2*, broader sequencing studies have identified additional co-mutations involving genes such as *ASXL1* and *SF3B1*, reinforcing the concept that VEXAS exists within a wider continuum of clonal hematopoiesis and myeloid dysregulation rather than as an isolated monogenic inflammatory disorder [2].

Studies by Sakuma et al. and Sirenko et al. further underscore the genomic heterogeneity of *UBA1*-mutant disease by expanding the non-Met41 UBA1 spectrum *(UBA1*^non-M41^*)* and documenting diverse co-mutation patterns across hematologic contexts [12,13,14]. Notably, Sakuma et al. demonstrated through functional testing of 18 UBA1 variants that *UBA1*^M41^ mutations exhibit limited co-mutational tolerance and associate predominantly with MDS, whereas *UBA1*^non-M41^ co-occur more frequently with additional myeloid gene mutations and span a broader range of hematologic neoplasms [13]. Sirenko et al. identified a 2% prevalence of likely pathogenic *UBA1* variants in a large MDS cohort (*n* = 2027), with *TET2* and *DNMT3A* as the most common co-mutations, and found that 82% of evaluable cases had VEXAS-associated diagnoses or inflammatory presentations [14]. These observations support a model in which co-mutations reflect the broader clonal milieu and disease context, while *UBA1* remains the defining driver.

## 6. Genotype–Phenotype Correlations

A somatic *UBA1* mutation is required for the diagnosis of VEXAS syndrome; however, additional genetic and biologic factors may influence clinical expression. These include non-M41 mutations, co-occurring clonal hematopoiesis-associated mutations, and relative clonal burden. As discussed above in the largest clonal hematopoiesis analysis to date, Gutierrez-Rodrigues et al. found that typical CH co-mutations *DNMT3A* and *TET2* were not associated with specific inflammatory or hematologic manifestations but were independently correlated with poor overall survival, alongside transfusion-dependent anemia and moderate thrombocytopenia as separate prognostic factors [2]. These findings should be interpreted cautiously, as most available studies are retrospective, involve relatively small sample sizes, and vary in patient selection, sequencing strategies, and clinical endpoints. Recent data from Sakuma et al. (2025) demonstrate distinct biological differences between canonical *UBA1*^M41^ and *UBA1*^non-M41^ mutations, with M41 mutations generally not tolerating multiple co-mutations, while non-M41 mutations more commonly appear with co-mutations, and hematologic neoplasms other than MDS are described in association [13]. Ferrada et al. demonstrated that the canonical M41 mutation p.Met41Val is associated with the highest mortality risk, whereas p.Met41Leu has a less severe phenotype. The p.Met41Val variant is also associated with more intense systemic inflammation and accelerated progression to bone marrow failure [17] (Table 1). They also showed that VEXAS syndrome severity correlates with residual translation of the cytoplasmic *UBA1* isoform UBA1b [17].

Furthermore, the literature clearly indicates that the molecular landscape of VEXAS-associated MDS is fundamentally different from traditional MDS. VEXAS-MDS is dominated by CHIP-associated mutations (*DNMT3A*, *TET2*, and *ASXL1*) rather than the spliceosome mutations and *TP53* alterations that define high-risk traditional MDS [2,5,18]. The NCCN also notes that isolated mutations of *DNMT3A*, *TET2*, and *ASXL1* have less predictive value for myeloid neoplasm transformation compared to spliceosome or *RUNX1* mutations, which aligns with the low AML progression rate observed in VEXAS [18].

## 7. VEXAS Syndrome in Women

VEXAS syndrome was initially reported almost exclusively in men, aligning with its X-linked genetic basis and the necessity for a somatic mutation in *UBA1* that impacts the X chromosome [1]. Subsequent studies, however, have confirmed that women can also develop VEXAS syndrome, although reported cases remain relatively uncommon [5,19].

In female patients, disease development is thought to depend on biological processes that effectively result in functional monosomy of the X chromosome within hematopoietic cells. In the recent largest dedicated female VEXAS cohort to date, an international multicenter study included 12 women with genetically confirmed VEXAS [20]. Acquired X monosomy was the predominant mechanism, identified in 6 of 8 (75%) women who underwent karyotypic testing. In the same study, additional clonal mutations were present in 3/5 tested women. These findings suggest that while acquired X monosomy accounts for the majority of documented female cases, alternative mechanisms must exist in a meaningful minority. Possible explanations include age-associated skewing of X-chromosome inactivation, acquired loss of one X chromosome, or structural abnormalities involving the second X chromosome. In these scenarios, a somatic *UBA1* mutation can exert pathogenic effects similar to those observed in male patients [19]. As in men, the mutation is restricted to hematopoietic lineages, producing a mosaic pattern of myeloid involvement. Women with VEXAS syndrome exhibit clinical features similar to those seen in men, which include systemic inflammation, polychondritis, cutaneous vasculitis, cytopenias, and vacuolization of the bone marrow [19]. Women may experience longer diagnostic delays, likely due to reduced clinical suspicion and the historical view of VEXAS as primarily a male condition. Importantly, coexisting clonal hematopoiesis-associated mutations, such as those involving *DNMT3A* and *TET2*, have also been identified in female patients, indicating that biological sex does not mitigate the risk of clonal hematopoiesis [19]. Regarding whether the underlying X-chromosomal mechanism modifies prognosis or treatment response, current evidence does not demonstrate a difference, and clinical features, age at onset, *UBA1* mutation type, VAF, and mortality are comparable between sexes [20].

The recognition of VEXAS syndrome in women has important implications for both diagnosis and disease conceptualization. It underscores that the somatic mutation of *UBA1*, not gender, forms the molecular basis for this disorder and stresses the importance of evaluating VEXAS in women who show late-onset, persistent inflammatory diseases along with unexplained cytopenias or abnormalities in the bone marrow.

## 8. Therapeutic and Prognostic Implications

VEXAS is associated with high morbidity and mortality, and symptoms are frequently treatment-resistant. High-dose glucocorticoids are often effective for acute inflammatory control, but relapse during tapering is common. Janus kinase inhibitors and cytokine-directed therapies, including IL-6 and IL-1 blockade, have shown efficacy in some patients, although comparative trials are lacking. The strength of evidence varies considerably across these therapeutic approaches. Current therapies primarily target downstream inflammation and do not eradicate the mutant hematopoietic clone. The detection of co-mutations such as *DNMT3A* or *TET2* may identify patients with more complex clonal hematopoiesis who warrant closer hematologic evaluation, but their role in guiding treatment decisions remains uncertain [5,21,22].

The NCCN MDS guidelines (Version 3.2026) list *UBA1* among genes somatically mutated in MDS at an estimated incidence of ~5%, with a footnote recommending rheumatology consultation and JAK2 inhibitors and/or hypomethylating agents (HMAs) may be used [18]. In the largest retrospective azacitidine (AZA) study to date, Jachiet et al. reported molecular response (≥25% reduction in *UBA1* VAF) was achieved in 65% of patients, with ~50% reaching VAF < 2% [23]. This study showed that AZA yields durable inflammatory, hematologic, and molecular responses in VEXAS syndrome, but the high relapse rate after discontinuation indicates a need for prolonged therapy to sustain disease control [23].

The Sakuma et al. review in *Oncotarget* explores clone-targeting therapies, including azacitidine (approved for MDS), novel UBA1 inhibitors being developed for cancers, PERK (protein kinase RNA-like endoplasmic reticulum kinase) inhibitors, and auranofin (a rheumatoid arthritis drug) [24]. Current data are insufficient to identify associations between *DNMT3A/TET2* mutations and response to hypomethylating agents. Allogeneic stem cell transplantation remains the only potentially curative approach; however, access is limited by the advanced age, comorbidities, and reduced performance status typical of VEXAS patients [5,21]. All available transplant data are retrospective—the largest series by Ali and Gurnari reported 81.8% overall survival among 33 transplanted patients, though graft-versus-host disease (GVHD) (56.3%) and infections (37.5%) were frequent complications, and conditioning regimens and GVHD prophylaxis platforms varied considerably [25]. A phase II prospective trial (NCT05027945) is ongoing to better define optimal conditioning, GVHD prophylaxis, and outcomes in this population [5].

## 9. Conclusions

VEXAS syndrome introduces a new framework for understanding inflammatory disease, in which somatic mutations arising in hematopoietic stem and progenitor cells lead to systemic autoinflammation. *UBA1* remains the essential driver mutation, and loss of function affecting the cytoplasmic E1 ubiquitin-activating enzyme provides a mechanistic link between innate immune activation and bone marrow pathology. Beyond canonical *UBA1* p.Met41 variants, VEXAS exhibits an expanded genomic landscape that includes non-Met41 mutations and co-occurring clonal hematopoiesis-associated alterations. These features contribute to molecular heterogeneity; while robust genotype–phenotype correlations remain incompletely defined, emerging evidence raises the possibility that *UBA1*^non-M41^ variants are associated with differences in clinical presentation, whereas co-occurring CHIP mutations may have greater relevance for disease outcomes and overall survival [2,5,12,13]. Even among canonical *UBA1*^M41^ variants, the available data suggest possible differences in disease severity between substitutions [17].

Sirenko et al. identified *UBA1* mutations in approximately 2% of MDS patients overall and 7% of males lacking typical MDS-defining mutations, with 82% having VEXAS-associated inflammatory manifestations in *UBA1* mutations [14]. These patients showed a distinct molecular profile with predominantly CHIP-associated mutations (*DNMT3A*, *TET2*, and *ASXL1*) rather than high-risk MDS mutations, and most were classified as lower-risk MDS with rare progression to AML [14]. Using stringent diagnostic criteria that exclude vacuolization alone as dysplasia, the true MDS incidence in VEXAS was only 18%, lower than the previously reported 18–55% range in earlier literature [5,14,21]. The NCCN guidelines now recognize *UBA1* mutations in approximately 5% of MDS cases and include *UBA1* in standard MDS molecular panels but do not provide specific targeted screening criteria. Complementing the NCCN framework, Sirenko et al. recommend systematic screening in male patients with unclassifiable MDS; clonal cytopenias of undetermined significance (CCUS) harboring isolated *DNMT3A*, *TET2*, or *ASXL1* mutations; concurrent inflammatory symptoms; or macrocytic anemia with bone marrow vacuolization [14]. The ACR guidance statement emphasizes multidisciplinary evaluation and baseline bone marrow examination for all cytopenic VEXAS patients [21]. Next-generation sequencing (NGS) and Sanger sequencing are both effective methods for detecting UBA1 mutations. In a cohort of 104 patients, von Bornemann Fløe et al. demonstrated that Sanger sequencing identified all 12 UBA1-mutant cases, with NGS detecting no additional VEXAS cases beyond those found by Sanger, supporting its use as a practical first-line test given its faster turnaround and lower cost [26]. NGS offers advantages in detecting low-level variants (<20% VAF), identifying non-canonical mutations outside exon 3, and assessing co-occurring myeloid mutations; many centers now routinely include UBA1 in myeloid malignancy NGS panels [18,21]. NGS may therefore be reserved for patients with high clinical suspicion of VEXAS despite a negative Sanger result or when concurrent myeloid mutation profiling is desired. Given that a majority of *UBA1*-mutated MDS patients had VEXAS-associated inflammatory manifestations, multidisciplinary evaluation is essential, with baseline bone marrow examination, cytogenetic analysis, and NGS or Sanger sequencing recommended for all cytopenic VEXAS patients [14,18,21,26].

The clinical vignette presented in this review illustrates several key concepts: the patient harbored a canonical p.Met41Leu UBA1 variant—reported to be associated with a less severe phenotype and better prognosis compared to p.Met41Val—yet experienced a severe disease course with progressive cytopenias, recurrent thromboses, and treatment-refractory inflammation. Notably, the co-occurring *DNMT3A* mutation was identified before *UBA1* testing, contributing to diagnostic delay and highlighting that even ‘milder’ *UBA1* genotypes can lead to significant morbidity when arising within a complex clonal hematopoietic background.

From a clinical perspective, early recognition of *UBA1* mutations can reduce diagnostic delay, while careful assessment of coexisting clonal hematopoiesis may inform hematologic monitoring and multidisciplinary management. An important unresolved priority and area for future research is the development of standardized genomic reporting for VEXAS. Although UBA1 VAF may have potential relevance for diagnosis, longitudinal monitoring, clonal dynamics, and treatment-response assessment, no validated VAF thresholds currently exist for clinical decision-making. Similarly, consensus is needed regarding which co-occurring clonal hematopoiesis-associated mutations should be routinely reported on VEXAS-focused NGS panels, particularly given their possible prognostic relevance and implications for hematologic surveillance. Future prospective studies integrating genomic, clinical, and functional data will be essential to clarify the biologic significance of co-mutations and to guide precision-based therapeutic strategies for this serious and often life-threatening condition.

## Figures and Tables

**Table 1:** UBA1 mutation types, clinical phenotypes, and prognosis in VEXAS syndrome.

Mutation Category	Specific Variants	Clinical Phenotype/Severity	Associated Hematologic Disorders	Co-Mutation Pattern	Prognostic Implications	References
**Canonical M41 Mutations**	p.Met41Val	Most severe phenotype; intense systemic inflammation; accelerated bone marrow failure; transfusion-dependent anemia; highest mortality risk	Classic VEXAS with MDS (18–55% depending on criteria)	Generally do not tolerate multiple co-mutations; lower co-mutation complexity	Worst prognosis among M41 variants	[13,14,17]
	p.Met41Thr	Intermediate severity: moderate thrombocytopenia, inflammatory eye disease, and ear chondritis	Classic VEXAS with MDS	Lower co-mutation complexity	Intermediate prognosis	[13,17]
	p.Met41Leu	Less severe phenotype; milder inflammation	Classic VEXAS with MDS	Lower co-mutation complexity	Better prognosis among M41 variants	[13,17]
**Non-M41 Mutations**	Various (e.g., other missense)	Distinct biologic and hematologic features; different patterns of marrow involvement	Broader spectrum: lymphomas, plasma cell dyscrasias, hematologic neoplasms beyond MDS	More commonly appear with multiple co-mutations; greater co-mutation complexity	Variable; associated with diverse hematologic malignancies	[13]
**Co-Occurring CH Mutations**	*DNMT3A*, *TET2*, *ASXL1*	Not associated with specific inflammatory or hematologic manifestations	CHIP-associated; low AML progression rate	Present in ~60% of VEXAS patients *; may arise before or after UBA1	Independently correlated with poor overall survival	[2,5]

* Cohort size n = 80.

## Data Availability

No new data were generated or analyzed in this study.
